# A set of defined oncogenic mutation alleles seems to better predict the response to cetuximab in CRC patient-derived xenograft than KRAS 12/13 mutations

**DOI:** 10.18632/oncotarget.5886

**Published:** 2015-10-26

**Authors:** Dawei Chen, Xuesong Huang, Jie Cai, Sheng Guo, Wubin Qian, Jean-Pierre Wery, Qi-Xiang Li

**Affiliations:** ^1^ Crown Bioscience, Inc., 3375 Scott Blvd, Santa Clara, CA 95054, USA; ^2^ State Key Laboratory of Natural and Biomimetic Drugs, Peking University, Beijing 100191, China

**Keywords:** biomarker, PDX, KRAS, erbitux, patient stratification

## Abstract

Cetuximab is a standard of care for treating EGFR-expressing metastatic colorectal carcinoma (mCRC) exclusive of those with KRAS mutations at codons 12/13. However, retrospective analysis has recently suggested that KRAS-G13D patients can still benefit, while only a fraction of KRAS wild-type patients can benefit, from the treatment. We set out to test this contradicting issue experimentally in an independent cohort of patient derived xenograft (PDX) diseases. We conducted a mouse clinical trial (MCT) enrolling a random cohort of 27 transcriptome sequenced CRC-PDXs to evaluate cetuximab activity. The treatment responses were analyzed against the KRAS 12/13 mutation alleles, as well as several other well-known oncogenic alleles. If the response is defined by >80% tumor growth inhibition, 8/27 PDXs (∼30%) are responders versus 19/27 non-/partial responders (∼70%). We found that indeed there are no significantly fewer KRAS-12/13-allele responders (4/8 or 50%) than non-/partial responders (7/19, or 37%). In particular, there are actually no fewer G13D responders (4/8, or 50%) than in non-/partial responders (2/19 or 10.5%) statistically. Furthermore, majority of the non-/partial responders tend to have certain activating oncogenic alleles (one or more of the following common ones: K/N-RAS-G12V/D, -A146T, -Q61H/R, BRAF-V600E, AKT1-L52R and PIK3CA-E545G/K). Our data on an independent cohort support the recent clinical observation, but against the current practiced patient stratification in the cetuximab CRC treatment. Meanwhile, our data seem to suggest that a set of the six-oncogenic alleles may be of better predictive value than the current practiced stratification, justifying a new prospective clinical investigation on an independent cohort for confirmation.

## INTRODUCTION

Colorectal cancer (CRC) is one of the most common and deadliest malignancies, with high frequency of metastasis (mCRC) (∼50%). The common treatment options include combination of different chemotherapy agents (*e.g*. 5-fluorouracil (5-FU)/leucovorin (LV)(IV), fluoropyrimidines (capecitabine, uracil/ftorafur) (UFT, oral), 5-FU/oxaliplatin (OX)/Leucovorin (FOLFOX), capecitabine/OX (CAPOX), capecitabine/irinotecan (CAPIRI) and FOLFIRI, (5FU/irinotecan)) and targeted Agents (e.g. bevacizumab (Avastin^®^), cetuximab (Erbitux^®^) [1, 2] and panitumumab (Vectibix^®^). The latter two are monoclonal antibodies targeting epidermal growth factor receptor (EGFR), which offer further clinical benefit for a subset of mCRC [3, 4]. Cetuximab was first approved by the US Food and Drug Administration (FDA) for treating EGFR-expressing mCRC, either as a single agent (for irinotecan-/OX-refractory patients) or in combination with irinotecan (for irinotecan-refractory patients) [1], excluding those with KRAS mutations at codons 12/13 [5]. However, only ∼10% of mCRC patients would respond to cetuximab monotheray as the second line therapy [6]. Reports have suggested that gene amplification and over-expression of EGFR or its ligands, epiregulin (EREG) and amphiregulin (AREG), could potentially serve as the positive predictors of the response [5, 7], while other genetic alterations could serve as negative predictors [5], including the activating mutations [2] of EGFR and BRAF (e.g. V600E), and the activation of ERBB2 signaling [8], in addition to the KRAS mutations [2, 9–12]. Nevertheless, with conflicting and inconclusive observations so far, it remains a challenge to predict the responders; and KRAS mutation still is the only biomarker used in patient stratification in the clinic.

Importantly, two pieces of evidences indicate that the current cetuximab label regarding KRAS mutation may be incorrect, at least inaccurate. First, a recent retrospective analysis of multiple phase-III trials unexpectedly concluded that patients with KRAS codon 13 mutation (G13D) seem to benefit from the treatment [13, 14]. Second, only 35∼50% wild-type KRAS CRC patients responded to cetuximab [2, 10], or nearly 50% false positive rate. Therefore, there is apparent medical importance and urgency to include previously excluded “responders” so they can benefit from the treatment and also to exclude the previously included “non-responders” to avoid their unnecessary cost and suffers.

Patient derived xenograft (PDX) mirrors patient tumors' histopathology and molecular pathology (“patient avatar”) [15–20], particularly those of metastatic tumors [15], and recently also in CRC [21]. Large panel of PDXs can reflect patient diversity and be used to evaluate therapy by modeling clinical trial format — “mouse clinical trial” or MCT [22]. This report described the establishment and molecular characterization of a large panel of CRC-PDXs. We conducted a MCT that was designed to experimentally test the roles of KRAS mutations, particularly G13D, along with other activating oncogene alleles in responses to cetuximab, in which a random cohort of 27 EGFR-expressing subjects were enrolled. Our data confirmed that KRAS wild type at positions 12/13 indeed is not predictive of response to cetuximab and that G13D not predictive of non-response. Rather, a small number of well-known oncogenic mutation alleles seem to have better predictive power than KRAS mutation at positions 12/13.

## RESULTS

### Genomic profiling of CRC-PDXs

We set out to establish CRC-PDXs and evaluate their response to cetuximab, and investigate biomarkers predictive of the response. CRC are among the cancer types that are most readily engrafted into immunocompromised mice with high take-rate [21] and we have successfully established a large collection of CRC-PDXs by subcutaneously transplanting unsupervised tumor tissues that were surgically removed from treatment-naïve Asian CRC patients. We next performed transcriptome sequencing (RNAseq) of these models and identified the oncogenic mutation alleles frequently found in CRC as listed in Table 1, including KRAS, NRAS, AKT1, BRAF, PIK3CA, and majority of the mutations were also confirmed by hot-spot mutation analyses. All the 27 PDXs express EGFR at mRNA levels, as shown in Supplementary Table 1. 15 PDXs contain KRAS mutations (56%), slightly higher than the reported 32–40% in patients. However, the deviation could result from higher take-rate of tumors with KRAS mutations, or simply small sample size. Among the 15 KRAS mutants, 5/15 are at codon-12 (G12C/D/V) (∼3%), 6/15 at codon-13 (G13D) (40%), 2/15 at Q61H (33%), and 2/15 at A146T (∼13%). In total, there are 73% mutations at codon 12/13, which is slightly lower than the clinically reported 85–90%. Mutations at codon 61 and 146 are more frequent than reported in the clinic (vs. 5% and 5%). Again, the engraftment may not necessarily favor theses non-codon-12/13 mutations because of small sample size. One out of the 27 PDXs harbors NRAS Q61R mutation (CR1574) (3.7%). Two out of the 27 models harbor BRAF V600E mutations (7.4%, CR0004 and CR0029), lower than the reported 15% in patients [23]. The BRAF mutations are mutually exclusive to KRAS mutations in these models as reported in CRC patients [2]. 5/27 with PIK3CA E545G/K mutations (4 E545K, 1 E545G), and 1/27 with AKT1 mutation (CR1744:L52R) (Table 1). In addition, several matched patient samples have also been analyzed for oncogene mutations, confirming the same genetic profiles (e.g. CR0455, Table 1) as seen by others [15]. We also confirmed that all the tested CRC-PDXs express EGFR at protein levels using IHC (examples shown in Supplementary Figure 1, and all summarized in Supplementary Table 1), as one of the current criteria for cetuximab treatment in the clinic, although there is no correlation found between response and EGFR levels. In addition, the corresponding patient information and histopathology are summarized in Supplementary Table 1.

**Table 1:** Cetuximab sensitivity and genetic profile for 26 CRC PDX models

Response	Model ID	ΔT/ΔC%	AKT1L52R	BRAFV600E	KRAS G12D/V/C	KRAS A146T	KRAS Q61H	KRASG13D	NRASQ61R	PIK3CAE545G/KQ546L
**Responder**	**CR2110**	**−48%**	**WT**	**WT**	**WT**	**WT**	**WT**	**WT**	**WT**	**WT**
	**CR0231**	**−13%**	**WT**	**WT**	**WT**	**WT**	**WT**	**G13D**	**WT**	**WT**
	**CR2502**	**−9%**	**WT**	**WT**	**WT**	**WT**	**WT**	**WT**	**WT**	**WT**
	**CR0170**	**−7%**	**WT**	**WT**	**WT**	**WT**	**WT**	**WT**	**WT**	**WT**
	**CR0196**	**−6%**	**WT**	**WT**	**WT**	**WT**	**WT**	**WT**	**WT**	**WT**
	**CR2520**	**1%**	**WT**	**WT**	**WT**	**WT**	**WT**	**G13D**	**WT**	**WT**
	**CR0588**	**11%**	**WT**	**WT**	**WT**	**WT**	**WT**	**G13D**	**WT**	**WT**
	**CR0193**	**16%**	**WT**	**WT**	**WT**	**WT**	**WT**	**G13D**	**WT**	**WT**
**Non-/partial responder**	**CR0047**	**27%**	**WT**	**WT**	**G12C**	**WT**	**WT**	**WT**	**WT**	**E545K**
	**CR0560**	**28%**	**WT**	**WT**	**WT**	**WT**	**WT**	**WT**	**WT**	**WT**
	**CR0205**	**34%**	**WT**	**WT**	**WT**	**WT**	**WT**	**WT**	**WT**	**WT**
	**CR0150**	**43%**	**WT**	**WT**	**G12D**	**WT**	**WT**	**WT**	**WT**	**WT**
	**CR1530**	**52%**	**WT**	**WT**	**WT**	**WT**	**Q61H**	**WT**	**WT**	**E545K**
	**CR2226**	**62%**	**WT**	**WT**	**WT**	**WT**	**WT**	**G13D**	**WT**	**WT**
	**CR1519**	**67%**	**WT**	**WT**	**WT**	**WT**	**Q61H**	**WT**	**WT**	**WT**
	**CR0245**	**69%**	**WT**	**WT**	**WT**	**A146T**	**WT**	**WT**	**WT**	**WT**
	**CR1554**	**69%**	**WT**	**WT**	**G12V**	**WT**	**WT**	**WT**	**WT**	**WT**
	**CR0004**	**75%**	**WT**	**V600E**	**WT**	**WT**	**WT**	**WT**	**WT**	**E545K**
	**CR1245**	**76%**	**WT**	**WT**	**G12D**	**WT**	**WT**	**WT**	**WT**	**WT**
	**CR0012**	**81%**	**WT**	**WT**	**WT**	**WT**	**WT**	**G13D**	**WT**	**WT**
	**CR0455**	**86%**	**WT**	**WT**	**G12D**	**WT**	**WT**	**WT**	**WT**	**WT**
	**CR1574**	**88%**	**WT**	**WT**	**WT**	**WT**	**WT**	**WT**	**Q61R**	**Q546L**
	**CR1795**	**94%**	**WT**	**WT**	**WT**	**WT**	**WT**	**WT**	**WT**	**WT**
	**CR0029**	**95%**	**WT**	**V600E**	**WT**	**WT**	**WT**	**WT**	**WT**	**WT**
	**CR0146**	**118%**	**WT**	**WT**	**WT**	**WT**	**WT**	**WT**	**WT**	**E545G**
	**CR1744**	**129%**	**L52R**	**WT**	**WT**	**WT**	**WT**	**WT**	**WT**	**WT**
	**CR0010**	**158%**	**WT**	**WT**	**WT**	**A146T**	**WT**	**WT**	**WT**	**E545K**

### KRAS 12/13 mutations not predictive of poor response to cetuximab

**Figure 1:**
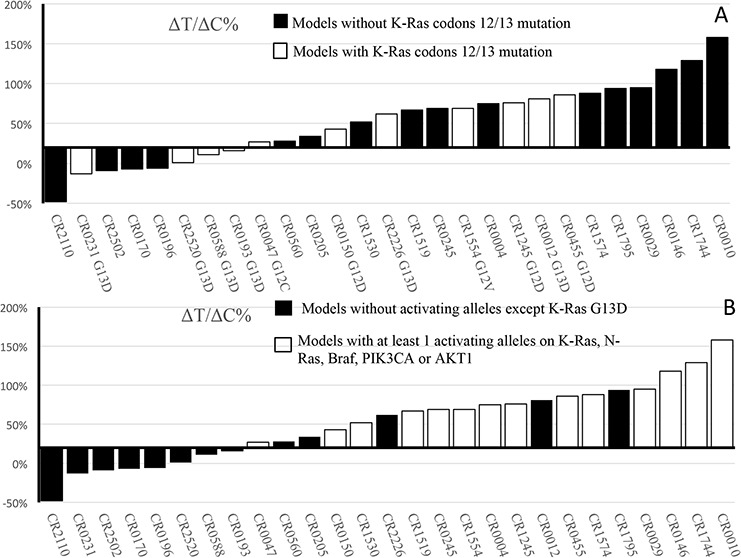
Waterfall plot of ΔT/ΔC% values of CRC-PDXs **A.** Per KRAS codons 12/13 mutation rule — wild type vs. mutations. **B.** Per the set of oncogenic allele rule –wild-type/KRAS-G13D vs. at least 1 activating alleles on KRAS-G12G12C/D/V, -Q61X, -A146T, NRAS-Q61X, AKT1-L52R, PIK3CA-E545K/-Q546L and BRAF-V600E.

Recent retrospective analysis of clinical data suggested that KRAS mutant at codon 13 can still benefit from the cetuximab treatment, contrasting to FDA guidance [2]. In order to investigate this further, we conducted a prospective mouse clinical trial by randomly enrolling a cohort of 27 experimental surrogate test subjects as listed in Table 1, CRC-PDXs, which have been transcriptome sequenced and confirmed to express EGFR. We subjected this cohort to cetuximab treatment, and the response to the treatment is analyzed using 20% ΔT/ΔC value as the threshold where lower value considered as responders and higher value considered non-/partial responders. The results demonstrated that 8/27 are responders (∼30%) and 20/27 are non-/partial responders (70%) (Table 1). The representative tumor growth inhibition of both responders and non-responders is shown in Supplementary Figure 1. Interestingly, G12C/D/V and G13D mutations are similarly found in both responders and non-responders (Table 1 and Figure 1A, Fisher's exact test *P* = 0.67 if only considering codon-12/13; *P* = 1.0 if considering all KRAS mutations), suggesting lesser roles of KRAS mutation in determining response than originally believed. In particular, there are 4/6 G13D falling into responders, while none for the non-G13D KRAS mutations (0/9), suggesting that indeed G13D patients can benefit from the treatment, while other KRAS mutation patients do not. This observation is consistent to the recent analysis of clinical observation [13, 14]. Considering that our data is completely independent of previous analysis (unrelated subjects and test methods), the observation is more likely to be true. It has been known that not all KRAS mutations are equal with regard to their activity and oncogenicity [14], which is strongly supported by our data.

### Certain oncogenic alleles better predictive of cetuximab response in CRC

The 5/5 G12C/D/V are all non-responders, 2/2 A146T (CR0010 and CR0245) and 2/2 Q61H (CR1515 and CR1530) are all non-responders. 1/1 NRAS Q61R is a non-responder (CR1574), mutually exclusive to KRAS mutation and BRAF mutation. Both BRAF-V600E containing models, CR0004 and CR0029, are non-responders (2/2) (Table 1 and Supplementary Figure 2), consistent to the observation that BRAF-V600E causes resistance to cetuximab [24] and is mutually exclusive to KRAS mutation. CR1744 with AKT1-L52R mutation is a non-responder (1/1), mutually exclusive to KRAS, NRAS, and BRAF mutation. 5/5 PIK3CA-E545K/Q546L mutants (exon 9) are all non-/partial responders, not mutually exclusive to other oncogene alleles, suggesting a possibly role of PIK3KCA mutations in cetuximab resistance [25], although not statistically significant (*P* = 0.28, Fisher's exact test).

In summary, 16/19 non-/partial responders have at least one of the activating alleles of KRAS-G12G12C/D/V (5/19), -Q61X (2/19), -A146T (2/19), NRAS-Q61X (1/19), AKT1-L52R (1/19), PIK3CA-E545K/-Q546L (5/19) and BRAF-V600E (2/19) (Table 1). This is in contrast to that 0/8 tested responders are wild-type for all these alleles (Fisher's exact test *P* = 7.43 × 10^−5^). Apparently, there are 5 models (5/19), CR-0560, −0205, −1795, −0012, −2226, where cetuximab resistant alleles are still yet to be identified [26]. This suggests that the composite oncogenic alleles profile could be more predictive. We should point out that the validity of this set of oncogenic alleles for predicting cetuximab resistance need to be further validated by testing in an independent cohort using a prospective design.

## DISCUSSION

Although KRAS-G13D has been suggested not used as predictor for poor response to cetuximab per recent retrospective review of past clinical data [13], it is still insufficient to change cetuximab label to recommend these patients for cetuximab treatment. Usually, only a confirmation in a prospective study using independent cohort of similar disease can potentially be used to change the label. Such a study is still to be conducted. PDXs have very similar histopathology and molecular pathology as patient tumors, and are thus considered closest surrogate experimental models for human tumors [15–19]. A cohort of diverse CRC-PDXs can be particularly useful to add a confirmation in a similar clinical trial as in human. Our prospective mouse clinical trial (MCT), using an independent cohort of test subjects, confirmed that G13D indeed cannot predict the poor response to cetuximab, in agreement with results from retrospective analysis of human data. This result further supports the notion that a human prospective trial should be conducted to confirm this and to change the label thus many G13D patients can also benefit from the treatment.

Our trial, using a random enrolled subjects, seems to discover a new set of oncogenic alleles, with only one of them being positive, to be better predictive of poor cetuximab response (no false positive, 38.5% false negative) than the current KRAS mutation at positions 12/13 (36.4% false positive, 75% false negative), or than all KRAS mutations (26.7% false positive, 66.7% false negative). On the other hand, the wild-type alleles/KRAS-G13D seem to be better predictive of response (38% false positive, 0% false negative) than the current wild type KRAS-12/13 rule (75% false positive, and 36.4% false negative). However, this proposed new biomarker signature derived from the current analysis needs further prospective clinical study, mouse and/or human, using independent cohorts, for confirmation. If confirmed, the label can be changed so that the patients with wild type KRAS mutation at position 12/13, but still with oncogenic alleles described in this report, should be excluded from the treatment so to avoid both unnecessary physical and economic harm to patients, and enable them to explore other treatment options.

PDX is an experimental model, although closely mimicking patient tumors. The observation derived from it still has to be proven in the clinics before clinical application. However, as an experimental model, it has certain advantages in scrutinizing the exact molecular mechanisms over the patients as testing subjects, including flexible design, precise enrollment (*e.g*. tumor size, enrolling time, *etc*.), subjects naïve to prior treatments, and precise dosing/pharmacokinetics, *etc*. Therefore, we advocate performing co-clinical trial of mouse and human for better understanding mechanism of drugs.

## MATERIALS AND METHODS

### Engraftment and tumor inhibition assays

The methods and the parameters regarding xenograftment of patient tissues and tumor inhibition assays using PDXs have been described previously [22, 23, 27]. Twenty-seven of these PDXs were randomly enrolled in *in vivo* tumor inhibition trial using cetuximab also as described [22, 23] (Crown Bioscience SPF facility). EGFR immunohistochemistry (IHC) analyses of model tumors was performed as previously described [22, 23].

### Genomic analysis of PDXs

For transcriptome sequence of PDX tumor tissues, per method described previously [22, 23], snap frozen samples were used to extract RNAs. The purity and integrity of the RNA samples were ensured by Agilent Bioanalyzer prior to RNA sequencing. Only RNA samples with RIN >7 and 28S/18S >1 were proceeded for library construction and RNA sequencing. RNA samples (mouse component <50%) were used for transcriptome sequencing by certified Illumina HiSeq platform service providers (BGI, Wuhan, China). The transcriptome sequencing was generally performed at 6GB, PE125 on Illumina HiSeq2500 platform or equivalent. For bioinformatics analysis of transcriptome sequencing data, RNAseq raw data was first cleaned up by removing contamination reads that preferentially mapped to mouse genome (UCSC MM9). Transcript expression was estimated by MMSEQ software, and the SNP/INDELs on the expressed genes were detected by BWA mapping software and GATK variant discovery toolkit, and the gene fusion was searched by SOAPfuse and Defuse. The confirmation of the hotspot mutation was conducted for some mutation alleles as previously described [23].

### Statistical analysis

We used Welch's *t*-test for two-sample comparisons, and one-way ANOVA for multiple-sample comparisons, and one-way ANOVA test for multiple comparisons as previously described [22, 23]. Fisher's exact test was used to assess the response difference between responders and non-responders.

## SUPPLEMENTARY TABLE AND FIGURES


