# Supplementary material for: A set of defined oncogenic mutation alleles seems to better predict the response to cetuximab in CRC patient-derived xenograft than KRAS 12/13 mutations
Source: Oncotarget. 2015 Oct 26;6(38):40815–21. doi: 10.18632/oncotarget.5886 (PMC4747370; doi:10.18632/oncotarget.5886)
Supplement: Supplementary file 1 [file oncotarget-06-40815-s001.pdf]

SUPPLEMENTARY TABLE AND FIGURES

Supplementary Table S1: Pathology diagnosis and EGFR expressions of PDXs

ID	Patient diagnosis	PDX diagnosis	EGFR RNAseq	EGFR IHC score
CR0004	ADC of colon, protruded type, moderately differentiated, invaded through intestinal wall to peripheral adipose tissue. Regional LN: paraintestinal LN (1/7). IHC results: HER-1(–), HER-2(–), p53(+ 50 ~ 75%), p170(–), Ki-67(+ 25 ~ 50%), VEGF(+++), Top-IIα(+<25%), p16(+<25%).	Moderately differentiated tubular ADC	2.56	2.5
CR0010	ADC of descending colon, carcinoma *in situ*, ulcerative type, well-moderately differentiated, tumor mass: 6 cm × 4.5 cm × 3.5 cm.	Moderately differentiated tubular ADC	1.69	3.0
CR0012	ADC of rectum, ulcerative type, carcinoma *in situ*, moderately differentiated, tumor mass: 6 cm × 5.5 cm × 1.5 cm.	Moderately differentiated tubular ADC	2.27	2.5
CR0029	Carcinoid, protruded type.	Poorly differentiated ADC	2.65	2.5
CR0047	NA	Moderately to poorly differentiated ADC	1.72	2.5
CR0146	ADC with mucinous carcinoma, moderately-poorly differentiated, ulcerative type, invaded serosa, tumor mass: 5 cm × 4 cm × 1cm. No malignant cells adjacent to both stumps. Regional LN: mesenteric LN (0/5).	Moderately differentiated tubular ADC	2.05	2.0
CR0150	ADC of cecum, moderately-poorly differentiated, ulcerative type, invaded serosa, tumor mass: 5 cm × 5 cm × 1c m. No malignant cells adjacent to both stumps. Regional LN: mesenteric LN (3/6).	Moderately to poorly differentiated ADC, 64-P4: Moderately differentiated tubular ADC	1.45	2.5
CR0170	ADC of rectum, ulcerative type, moderately-poorly differentiated, invaded deep muscular layer, tumor mass: 4 cm × 3 cm × 1cm. No malignant cells adjacent to both stumps. Regional LN: mesenteric LN (0/2).	Moderately to poorly differentiated ADC	2.03	2.0
CR0193	ADC of colon, moderately differentiated, ulcerative type, invaded serosa, tumor mass: 6.5 cm × 4.5 cm × 1.5 cm. No malignant cells adjacent to both stumps. Regional LN: mesenteric LN (0/9).	Moderately differentiated ADC	2.41	1.5
CR0196	ADC of the rectum, moderately differentiated, ulcerative type, invaded serosa, tumor mass: 3.5 cm × 3 cm × 0.8 cm. No malignant cells adjacent to both stumps. Regional LN: mesenteric LN (8/10).	Moderately differentiated ADC	2.25	2.5

(*Continued*)

ID	Patient diagnosis	PDX diagnosis	EGFR RNAseq	EGFR IHC score
CR0205	1. ADC of transverse colon, moderately-poorly differentiated, infiltrative type, invaded serosa, tumor mass: 8 cm × 7 cm × 1.5 cm. No malignant cells adjacent to both stumps. Regional LN: mesenteric LN (1/7).<br>2. ADC of duodenal papilla, moderately-poorly differentiated, invaded submucosa, tumor mass: 2.4 cm × 1.2 cm × 1cm. No malignant cells adjacent to both stumps and pancreatic stump. Regional LN: parahepatic artery LN (0/2), LN surround common hepatic duct (0/3), greater curvature LN (0/2), parapyloric LN (0/8), peripancreatic LN (0/1).	Moderately to poorly differentiated ADC	2.76	2.0
CR0231	NA	Moderately differentiated ADC with part of mucinous ADC	1.66	2.0
CR0245	ADC of rectum, ulcerative type, moderately differentiated, invade muscular layer to rectal serosa, tumor mass: 5 cm × 5 cm × 2 cm. No malignant cells adjacent to superior and inferior stump. Mesenteric LN (0/7).	Mucinous ADC	2.07	2.5
CR0455	ADC of colon, poorly differentiated.	Poorly differentiated ADC	1.66	2.5
CR0560	NA	Moderately to poorly differentiated ADC	1.87	2.5
CR0588	NA	Poorly differentiated squamous cell carcinoma	1.51	1.5
CR1245	ADC of right lung, moderately differentiated (mixed type, include acinic cell ADC and mucinous ADC).	ADC	1.79	
CR1519	NA	Poorly differentiated ADC	1.72	1.5
CR1530	NA	Moderately differentiated ADC	1.74	3.0
CR1554	ADC of rectum, ulcerative type, moderately differentiated, invade muscular layer to rectal serosa, tumor mass: 5 cm × 5 cm × 2 cm. No malignant cells adjacent to superior and inferior stump. Regional LN: mesenteric LN (0/7).	Moderately to poorly differentiated ADC with part of normal intestinal mucosa<br>56-P5: Moderately differentiated ADC	1.90	2.0
CR1574	NA	Mucinous ADC	2.72	1.0
CR1744	Carcinoma of the gingiva. IHC results: CK(+), Vim(+), P63(+), SMA(+), CD5/6(focus +), CD10(+)	Moderately differentiated ADC<br>60-P5: Mucinous ADC	2.23	1.5

(*Continued*)

ID	Patient diagnosis	PDX diagnosis	EGFR RNAseq	EGFR IHC score
CR1795	ADC, ulcerative type, moderately-poorly differentiated, invade through intestinal wall to adipose tissue. No malignant cells adjacent to both stumps. Regional LN: paraintestinal LN (0/27).IHC results: CK7(–), CK20(+), EGFR(++), P53(+), Ki-67(80%+), Villin(+++), CerbB-2(+), OCT3/4(–).	Poorly differentiated ADC	2.76	ND
CR2110	ADC of sigmoid colon, moderately-poorly differentiated, ulcerative type, tumor cell invade serosa and adhere small intestine, tumor mass: 6 cm × 4 cm. No malignant cells adjacent to superior and inferior stump. Regional LN: pericolic LN (0/16), LN around small intestine (0/4). IHC results: CD44(+), Cmet(+), COX2(+), EGFR(–), HER2(1+), Ki-67(+ <25%), MLH1(+50–75%), MMP7(+), MSH2(+>75%), MSH6(+ <25%), P170(+), P27(+25–50%), P53(+>75%), TOPOII(+<25%).	Moderately-poorly differentiated ADC	2.35	2.5
CR2226	Mucinous ADC of colon, protruded type, with part of moderately differentiated ADC, tumor mass: 6.5 × 3.5 cm. Malignant cells invade peripheral adipose tissue. Regional LN: paraintestinal LN (3/47), LN of ligamentum hepatoduodenale (0/1), LN of porta hepatis (0/5). IHC results: CD44(+), cMet(–), COX2(+), EGFR(–), HER2(1+), Ki67(+25–50%), MLH1(+50 ~ 75%), MMP7(weak +), MSH2(+<25%), MSH6(+50 ~ 75%), P170(+), P27(+<25%), P53(–), TOPOII(–).	Mucinous ADC	1.80	ND
CR2502	ADC of colon, protruded type, poorly differentiated, invaded peripheral adipose tissue. No malignant cells adjacent to both stumps. Regional LN: paraintestinal LN (5/17). IHC results: Syn(–), CHgA(–), NSE(–), CK7(–), CK20(++), Villin(+), CA199(++), CEA(++), P53(+), Ki67(80%+).	Poorly differentiated ADC	3.07	ND
CR2520	ADC of colon, protruded ulcerative type, moderately differentiated, invaded through intestinal wall. No malignant cells adjacent to both stumps. Regional LN: paraintestinal LN (0/26). IHC results:CD44(+), cMet(+), COX2(+), EGFR(+), HER2(1+), Ki-67(+>75%), MLH1(–), MMP7(+), MSH2(+50~75%), MSH6(+>75%), P170(+), P27(+<25%), P53(+<25%), TOPOII(+<25%).	Moderately differentiated ADC	2.17	2.0

Note: ADC: Adenocarcinoma

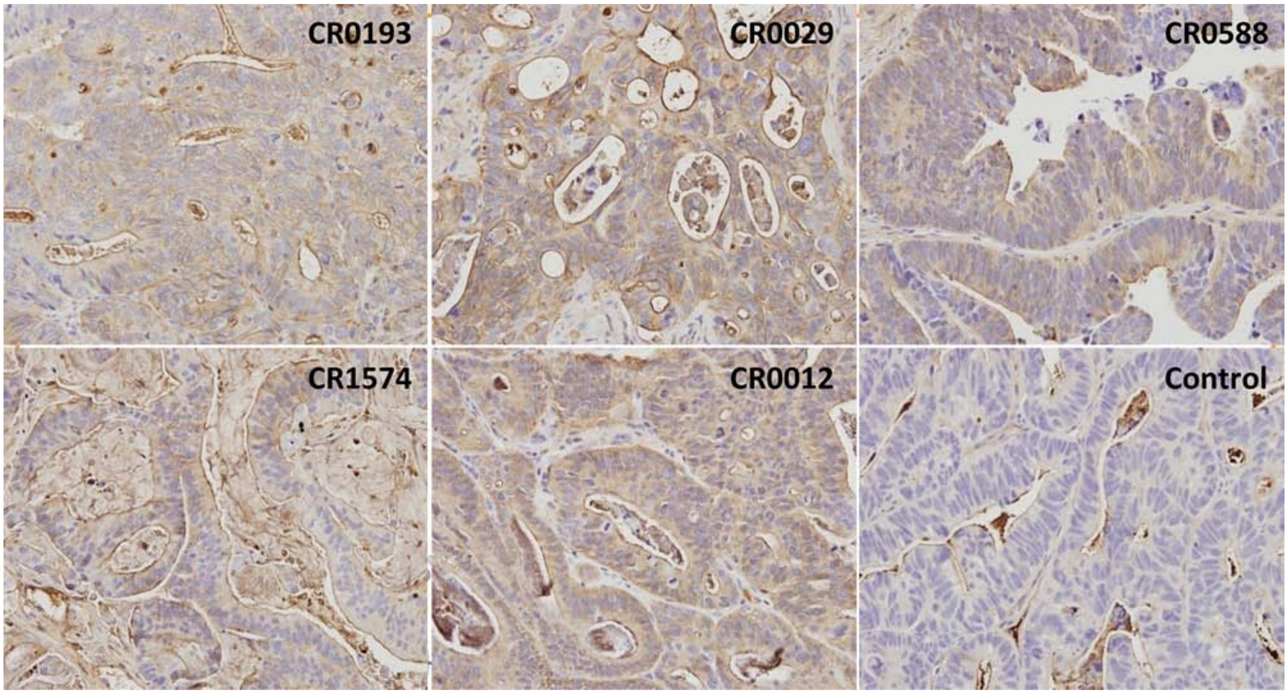

Supplementary Figure S1: EGFR-immunochemistry staining of CRC-PDX.

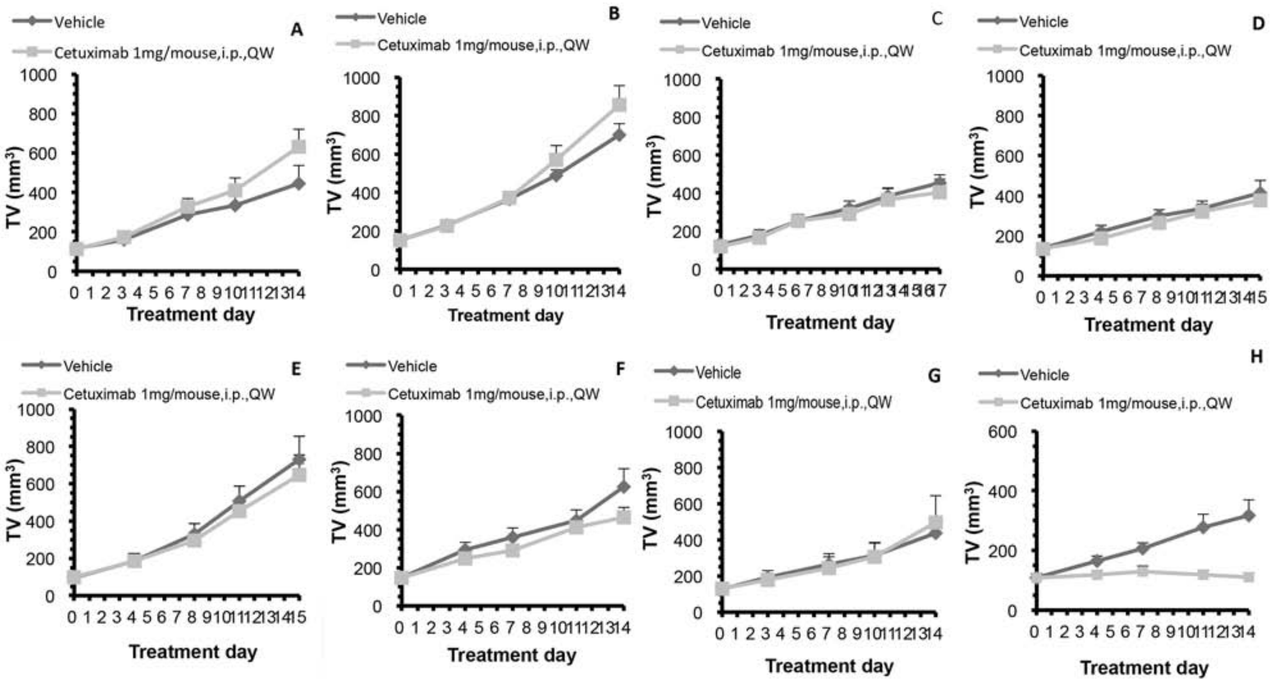

Supplementary Figure S2: Anti-tumor activity of cetuximab in 8 CRC-PDXs. Dark line: average tumor size (in 5 animals) for vehicle group (PBS qwX2 IP -- weekly for 2 weeks). Light line: average tumor size in treated group (5 mice), 1mg cetuximab qwX2 IP. **A.** CR0010: a non-responder with KRAS A146T mutation; **B.** CR1744: a non-responder with AKT1 L52R mutation; **C.** CR029: a non-responder with BRAF V600E mutation; **D.** CR1574 a non-responder with NRAS Q61R mutation. **E.** CR0455: a non-responder with KARS G12D mutation; **F.** CR1519: a non-responder with KRAS Q61H mutation. **G.** a non-responder with PI3K E545G mutation. **H**. a responder with KRAS G13D mutation.
